# NECAB1-3, parvalbumin, calbindin, and calretinin in the hippocampus of the European mole

**DOI:** 10.3389/fnana.2024.1452722

**Published:** 2024-09-04

**Authors:** Jovana Maliković, Irmgard Amrein, Lorenzo Vinciguerra, David P. Wolfer, Lutz Slomianka

**Affiliations:** ^1^Division of Functional Neuroanatomy, Institute of Anatomy, University of Zürich, Zürich, Switzerland; ^2^Department of Health Sciences and Technology, ETH Zürich, Zürich, Switzerland; ^3^Natural History Museum, St. Gallen, Switzerland

**Keywords:** immunohistochemistry, calcium-binding proteins, Eulipotyphla, comparative neuroanatomy, histoarchitecture, mossy cells

## Abstract

Many calcium-binding proteins are expressed in a region-and cell-type specific manner in the mammalian hippocampus. Neuronal calcium-binding proteins (NECABs) are also expressed in hippocampal neurons, but few species have been investigated, with partly controversial findings. We here describe NECAB1, NECAB2 and NECAB3 as well as parvalbumin, calbindin, and calretinin in the European mole, and compare staining patterns of these proteins with those in mouse and other species. While subtle differences are present, NECAB staining in the European mole was generally similar to those in mouse. Common to European moles, mice, and other species we investigated, large hilar polymorphic cells, likely to represent mossy cells, were positive for all three NECABs. NECAB1 and 2 are suitable as markers for these cells along the entire septotemporal axis of the hippocampus. In the European mole, parvalbumin, calbindin and calretinin showed traits that have been described in other species before, albeit in a unique combination. In summary, we provide the first description of distribution of these proteins in the hippocampus of the European mole. This subterranean, insectivorous, and solitary living species belongs to the Order of Eulipotyphla. Despite many similarities with other subterranean species from the rodent order in terms of lifestyle, its hippocampus is cytoarchitecturally much more elaborated than in, e.g., mole-rats. It remains an open question if the hippocampal structure of the European mole reflects evolutionary constraints or ecology. Our descriptive study highlights the diversity in hippocampal cytoarchitecture even in small mammalian species.

## 1 Introduction

Hippocampal neurons express a diversity of calcium-binding proteins, which are specific to regions, layers, and cell populations (Girard et al., 2015). Among them, calbindin, calretinin, and parvalbumin are extensively used as markers due to their relation to functionally defined neuronal subpopulations (Somogyi and Klausberger, 2005; Tzilivaki et al., 2023) and are valuable tools for the definition of hippocampal areas in comparative neuroanatomy (Seress et al., 1991; Seress et al., 1993; Murakawa and Kosaka, 1999; Slomianka et al., 2013; Pillay et al., 2021). More recently, a family of neuronal calcium-binding proteins (NECABs) has been described (Sugita et al., 2002; Zimmermann et al., 2013). Information on the immunohistochemical distribution of the NECABs within the hippocampal formation is fragmentary and sometimes inconsistent (Sugita et al., 2002; Zimmermann et al., 2013). So far, NECABs have only been evaluated in mice, rats and humans (Sugita et al., 2002; Maldonado-Avilés et al., 2006; Wu et al., 2007; Canela et al., 2009; Zimmermann et al., 2013; Miczán et al., 2021; Sanders et al., 2021). The three known members of the NECAB family exhibit structural similarities, with a single EF-hand domain for Ca^2+^ binding at the N-terminal end (Sugita et al., 2002). The binding of Ca^2+^ to the EF-hand domain may influence the activities of neighboring domains within the protein (Sugita et al., 2002). The precise functions of NECABs remain a subject of ongoing research, with hypotheses suggesting roles as either Ca^2+^-dependent activators of target proteins or Ca^2+^-buffers (Bernier et al., 2001; Sugita et al., 2002). NECAB sequences of mouse and human are remarkably similar (Sugita et al., 2002). Zimmermann et al. (2013) showed that the genes responsible for the three NECAB isoforms exhibit distinct expression patterns within specific subregions of the hippocampus, suggesting that, despite their structural resemblances, NECABs may serve distinct functional roles within the hippocampal formation.

Our aim was to map the distribution patterns of calbindin, calretinin, and parvalbumin, along with the more recently described NECAB1-3, in the European mole (*Talpa europaea*). This species was selected for multiple reasons. European moles belong, together with, e.g., shrews and hedgehogs (Douady and Douzery, 2003), to the mammalian order of Eulipotyphla (comprising many species of the now obsolete order of Insectivora). The ~68 million years since the split of Eulipothyphla from other mammalian orders (Álvarez-Carretero et al., 2022) provide ample time for diversification, which facilitates the definition of which changes in the distribution of neuronal markers may be possible over time. To this end, we will directly compare the distribution of NECABs with observations in C57BL/6 mice. Another reason is the similarity in lifestyle of European moles and mole-rats (Bathytherigidae), which belong to the order of Rodentia. Like mole-rats, European moles are strictly subterranean species, with sensory adaptations to the subterranean lifestyle. The mole eye, although structurally complete, is microphthalmic and concealed by fur, an external ear is missing and hearing is anatomically adapted to the detection of frequencies below 15 kHz (Kryštufek and Motokawa, 2018). Moles are solitary, a social structure also found among the socially diverse mole-rats. We previously found that the hippocampus of mole-rats differed in qualitative and quantitative traits from other rodents (Amrein et al., 2014; van Dijk et al., 2016), and we were interested in if similar traits could be observed in a phylogenetically quite different but ecologically similar species. Lastly, initial assessment of histoarchitecture and antigen distributions in the European mole showed a well differentiated, outright pretty (to us) hippocampus that begged for presentation.

Here, we provide a description of the European mole hippocampus cytoarchitecture along with the distribution patterns of six calcium-binding proteins and discuss the findings in comparison to the laboratory mouse and other mammalian species.

## 2 Materials and methods

### 2.1 Animals

One female and three adult male European moles (*Talpa europaea,* Talpidae, Eulipotyphla; body weight 50–64 g, brain weight 0.8–1 g) were obtained from field research mapping the biodiversity of small mammals in the Canton St. Gallen by the Natural History Museum of St. Gallen, Switzerland.

Four C57BL/6 J adult female mice were obtained from Charles River Laboratories (Germany). Permits were issued by the Office for Nature, Hunting and Fishing of the Canton of St. Gallen for European moles and from the Canton of Zurich Veterinary Office for laboratory mice (license ZH041/2018, #33353). Although previous studies did not note sex differences in the distributions of the calcium binding proteins assessed in this work (Celio, 1990; Holm et al., 1990; Liu et al., 1996; Slomianka et al., 2013; Zimmermann et al., 2013), the unbalanced sex distributions of the available specimens do not allow us to assess sex differences.

Brains of European moles were removed postmortem from the cranial cavities and immersion-fixed in a solution consisting of 4% phosphate-buffered paraformaldehyde (PFA) and 15% picric acid. Mice were deeply anesthetized, perfused transcardially with cold phosphate buffered saline (PBS, pH 7.4), followed by 4% PFA. Extracted brains were postfixed for 24 h in 4% PFA with 15% picric acid. One hemisphere from each animal was cryoprotected in a solution of 30% sucrose, frozen and cut horizontally at a thickness of 40 μm using a sliding microtome. Serial sections were collected and preserved in cryoprotectant at −20°C.

### 2.2 Immunohistochemistry

We used free-floating serial sections that spanned the entire hippocampus. Controls for non-specific binding of secondary antibodies were executed routinely by omitting the primary antibody. Staining procedures were adjusted, and antibody concentrations titrated for each species to obtain saturated staining of the strongest staining neurons. None of the antibodies exhibited staining in structures deemed nonspecific based on mouse sections processed in the same batch and previously published antigen distributions. Without knock-out controls for the European mole, all descriptions of antigen distributions should however be read as NECAB1-3-, calbindin-, parvalbumin-or calretinin-*like* immunoreactivities.

In addition to the immunohistochemical stains, a complete series of sections of each animal was mounted in the correct anatomical order, Nissl stained with Giemsa (Giemsa stock solution 1:5 in KH_2_PO_4_), dehydrated and coverslipped.

#### 2.2.1 NECABs

For antigen retrieval, sections of European mole were treated with 0.5% sodium borohydride in phosphate-buffered saline (PBS) for 30 min, while mouse sections were heat-treated in the microwave in citrate buffer (1:10 in dH2O, Sigma Aldrich) for one minute at 900W. In both species, endogenous peroxidase was blocked with 0.6% hydrogen peroxide in TBS-Triton (Tris buffered saline, 1:10 of Tris base in deionized H_2_0 + 0.05% of Triton, pH 7.4) for 15 min. Sections were preincubated in a solution of 2% normal goat serum and 0.25% of Triton in TBS for 1 h.

Sections were transferred into preincubation solution containing one of the primary antibodies: polyclonal rabbit anti-NECAB1 (Invitrogen, PA554849, Lot 37,970, RRID: AB_2644587, European mole: 1:1000, mouse: 1:300), polyclonal rabbit anti-NECAB2 (Atlas Antibodies, HPA013998, Lot 13,019, RRID: AB_1848016, European mole: 1:1000, mouse: 1:300) or polyclonal rabbit anti-NECAB3 (Atlas Antibodies, HPA071786, Lot R103584, RRID: AB_2732186, European mole: 1:1000, mouse: 1:300). The incubation time for the primary antibodies was overnight for the European mole and 3 days for mouse, both at room temperature.

Next, sections were washed in TBS. Secondary antibody incubation for both species (goat anti-rabbit, 1:300, Vector labs, BA-1000, Lot X11041) was performed in 2% normal goat serum and 0.1% BSA in TBS at room temperature for 40 min for the European mole and overnight for mouse. Finally, the sections were treated with ABC (Vector labs, Lot ZJ0909) in TBS, for 1 h (European mole) or 4 h (mouse), rinsed, and diaminobenzidine (DAB) stained. Following these steps, the sections were mounted onto gelatin-coated slides, dehydrated, cleared and coverslipped.

NECAB immunohistochemistry was also performed on various species of which spare sections were available from previous projects. NECAB staining of hilar polymorphic cells is illustrated here for Damaraland mole-rats (*Fukomys damarensis*, material from Amrein et al., 2014), guinea pig (*Cavia porcellus*) and domestic sheep (*Ovis aries*, material from Maliković et al., 2023 and Maliković, unpublished data), and eastern rock sengi (*Elephantulus myurus*, material from Slomianka et al., 2013).

#### 2.2.2 Calbindin, calretinin, and parvalbumin

The distributions of calbindin, calretinin and parvalbumin are well-documented in the laboratory mouse and other species. Only sections of the European mole were immunohistochemically processed for these calcium-binding proteins. Epitopes were retrieved by microwave treatment (1 min at 900 W) in citrate buffer (1:10 in dH_2_O, Sigma Aldrich). The endogenous peroxidase block was identical to the NECABs protocol. Subsequently, sections were preincubated for 1 h in 2% normal serum (calbindin and calretinin: goat, parvalbumin: horse) with 0.25% Triton in TBS. Next, sections were incubated overnight at room temperature using either polyclonal rabbit anti-calretinin (Swant, CR7697, Lot 1893–0114, RRID:AB_2619710), polyclonal rabbit anti-calbindin (Swant, CB-38a, Lot 9.03, RRID:AB_10000340) or monoclonal mouse anti-parvalbumin (Sigma Aldrich, P3088, Lot 100 M4797, RRID:AB_477329). A dilution of 1:1000 in preincubation solution was used for all antibodies. Thereafter, sections were washed in TBS and then incubated with goat anti-rabbit (1:300, Vector labs, BA-1000, Lot X11041, RRID:AB_2313606) or horse anti-mouse (1:300, Vector labs, BA-2000, Lot ZF0521, RRID:AB_2313581), diluted in TBS containing 2% serum of the host species plus 0.1% BSA, for 40 min at room temperature. After this step sections were incubated for 20 min with ABC in TBS (Vector Labs, Lot ZJ0909), stained with DAB, mounted and coverslipped.

### 2.3 Nomenclature

The nomenclature of hippocampal areas follows common use and is detailed in Maliković et al. (2023). In this work, the end of the mossy fiber zone (or stratum lucidum) marks the border between CA1 and CA3. Two definitions of CA2 (Lorente De Nó, 1934; Lein et al., 2005) are currently in use placing CA2 either proximal or distal to the end of the mossy fiber zone, and both definitions are being actively refined (Palomero-Gallagher et al., 2020; Lothmann et al., 2021; Bienkowski, 2023; Insausti et al., 2023; Radzicki et al., 2023; González-Arnay et al., 2024). Because of the ambiguity both within and across species and to maintain compatibility with earlier descriptions of calcium-binding proteins, we mostly avoid using the term CA2.

### 2.4 Imaging

Unless noted otherwise in the figure legends, images represent the mid-septotemporal hippocampal region and adjacent cortices in horizontal sections. Images were acquired with a Zeiss Imager.M2 microscope using the slide scanning mode of Stereo Investigator vers. 10 (MBF Bioscience, Williston, VT, RRID:SCR_002526) and x10 (final resolution of 1.5 pixels/μm) or x20 objectives (final resolution of 3 pixels/μm). The hippocampal region and adjacent cortices were masked, and contrast and brightness were adjusted globally to reflect the appearance of the sections when inspected by eye. No local image manipulations were performed. When possible, high magnification figures were generated from the scans presented in the low magnification figures. Other, mainly adjacent sections were used when a particular trait was not visible in the scans used for the low magnification figures.

## 3 Results

### 3.1 Cytoarchitecture of the European mole hippocampus

**Figure 1:**
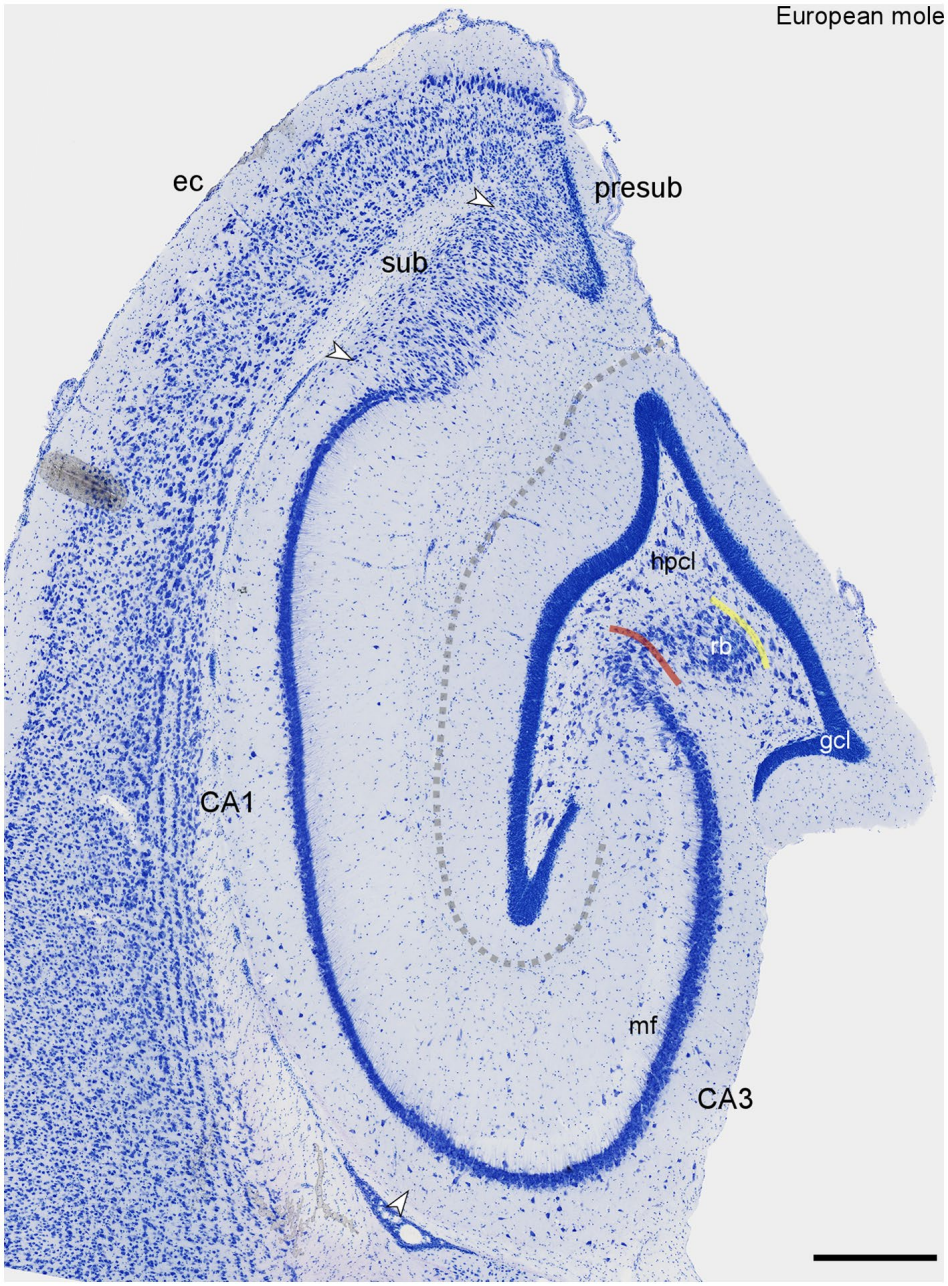
Histoarchitecture of the mid-septotemporal hippocampal region of the European mole. Arrowheads mark subfield boundaries between CA3, CA1, subiculum (sub) and presubiculum (presub). The yellow line marks the boundary between the hilar polymorphic cell layer (hpcl) and the reflected blade (rb) of CA3. The red line marks the border between the reflected blade and the remainder of CA3 in which the apical dendrites of the pyramidal cells point in the direction of the stratum lacunosum-moleculare of CA3 (Maliković et al., 2023). Similar to Nissl stains of other species (Maliković et al., 2023), the mossy fiber zone (mf) is visible as a light band also in the European mole. The dashed line marks the obliterated hippocampal fissure. This figure appears very similar to plate 11 of Rose (1912). Unfortunately, Rose did not provide further details. ec, entorhinal cortex. Scale bar: 500 μm.

The dentate gyrus is a prominent structure within the European mole hippocampus (Figure 1). The granule cell layer is sharply delimited from both the molecular layer and the hilus. Both the infra- and suprapyramidal blades may undulate, and they both consistently curve inwards, toward the CA3 pyramidal cell layer, close to their tips (Figure 1). A cell poor subgranular zone is present, separating the granule cells from a distinct hilar polymorphic cells layer (hpcl, Figure 1). The CA3 pyramidal cells form a loosely organized reflected blade (hpcl, Figure 1) within the curvature of the dentate gyrus. As brought out more clearly in sections stained for calcium-binding proteins (see below), the reflected blade is delimited from the hilar polymorphic cell layer by a continuation of the stratum radiatum of CA3. The remainder of CA3 forms a dense band composed of large pyramidal cells. In the deep pyramidal cell layer, large pyramids extend for a short distance beyond the tip of the mossy fiber zone (likely representing Lorente de No’s CA2). Thereafter, these cells are replaced by small, densely packed pyramids of CA1. CA1 is essentially of the compact type, with no or very little morphological differentiation of deep and superficial CA1 pyramidal sublayers (Figure 1). It is only close to the subiculum that the CA1 pyramidal cell layer may split into deep and superficial tiers (Figure 1). The transition to the subiculum is rather sharp. Proximally, the superficial subicular cell layer is dominated by pyramidal cells. The deep proximal subicular cell layer and its entire distal part are composed of morphologically more heterogenous cell populations (Figure 1). The transition to the presubiculum (Figure 1) is marked by distinct increases in cell density and decreases in cell size in the adjacent presubicular layers.

### 3.2 NECABs in European mole and C57BL/6J mouse

Unless specified, observations apply to both European mole and mouse. We found the distribution of NECABs to be stable along the septotemporal axis of the hippocampus. Unless noted otherwise, descriptions are applicable to the entire septotemporal axis.

#### 3.2.1 NECAB1

**Figure 2:**
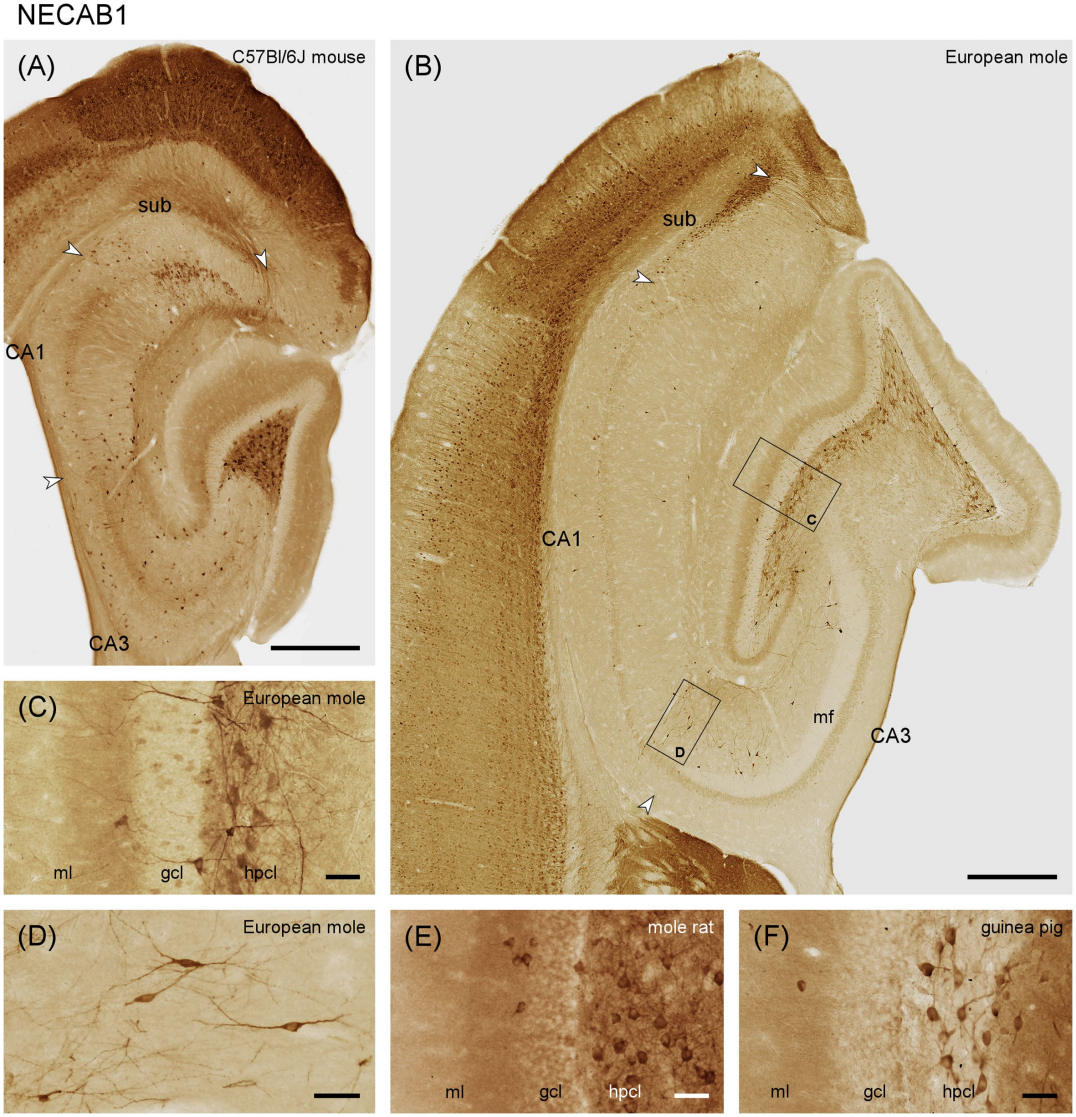
NECAB1 in the mid-septotemporal hippocampal region of the **(A)** C57BL/6 J mouse and **(B)** European mole. Arrowheads mark subfield boundaries between CA1 and CA3, CA1 and subiculum and subiculum and presubiculum. Black boxes mark the locations of **(C,D)**. Higher magnifications of NECAB1 **(C)** in the layers of the dentate gyrus showing the subset of stained granule cells, pyramidal basket cells and strongly stained multipolar cells in the hilar polymorphic layer in European mole. Bipolar neurons in stratum radiatum **(D)** of the European mole are well defined by NECAB1. NECAB1 in the layers of the dentate gyrus of the **(E)** Damaraland mole-rat and **(F)** guinea pig corroborate strong staining of hilar polymorphic cells in other species. gcl, granule cell layer; hpcl, hilar polymorphic cell layer; mf, mossy fiber zone; ml, molecular layer; so, stratum oriens; sr, stratum radiatum; sub, subiculum. Scale bars **(A,B)** 500 μm, **(C–F)** 50 μm.

Only the deep dentate molecular layer is moderately stained in European mole (Figures 2B,C). In mouse, both the middle and deep layers appear stained (Figure 2A). Rare interneurons are present in the deep molecular layer. A small subset of the granule cells is weakly stained in mole (Figure 2C), while all granule cells appear unstained in mouse. Strongly stained pyramidal basket cells are found along the border between the granule cell layer and the subgranular zone (Figure 2C). Prominent labeling is also observed in large multipolar cells, presumably hilar mossy cells, within the hilar polymorphic cell layer (Figures 2A–C). Strong immunoreactivity is seen in mostly bipolar interneurons throughout the stratum radiatum of CA3 (Figure 2D). Stained interneurons are present in stratum oriens of mouse (Figure 2A) but absent from mole (Figure 2B). The number of stained interneurons is generally lower in CA1 than in CA3, but a few cells are now also seen in stratum oriens of the mole (Figure 2B). In mouse, stained interneurons concentrate at the border between stratum radiatum and stratum lacunosum-moleculare (Figure 2A). In mole, frequent deep subicular cells display strong staining forming a compact layer (in particular distally) along the border toward the white matter (Figure 2B). In mouse, proximal superficial subicular cells are stained, alongside a subset of weaker stained deep distal cells. A few stained interneurons are found in the deep subicular plexiform layer (Figure 2A). We also observed NECAB1 staining of large hilar polymorphic cells in, e.g., Damaraland mole-rats (Figure 2E) or guinea pig (Figure 2F).

#### 3.2.2 NECAB2

**Figure 3:**
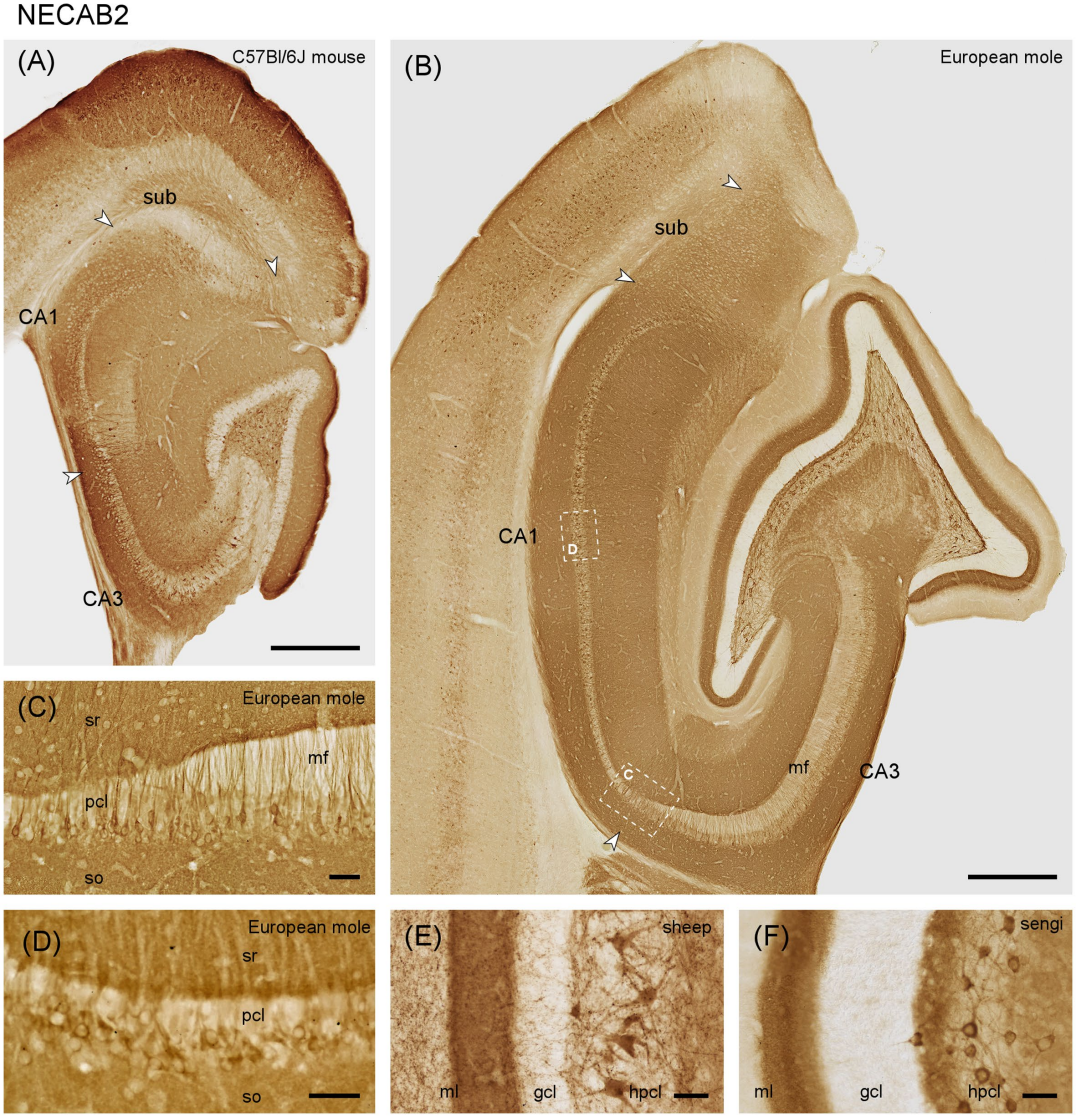
NECAB2 in the mid-septotemporal hippocampal region of the **(A)** C57BL/6 J mouse and **(B)** European mole. Arrowheads mark subfield boundaries. Dashed-bordered boxes mark the approximate locations of **(C,D)**, which were imaged in adjacent sections. NECAB2 in **(C)** distal CA3 and proximal CA1 pyramidal cells stains stronger than elsewhere in the pyramidal cell layer in European mole. Near perfectly perpendicular sections through the CA1 pyramidal layer **(D)** reveal NECAB2 in deep mid-proximodistal CA1 pyramidal cells in mole. NECAB2 in the layers of the dentate gyrus of the **(E)** domestic sheep and **(F)** eastern rock sengi again highlight the NECAB2 positive polymorphic cells in other species. gcl, granule cell layer; hpcl, hilar polymorphic cell layer; mf, mossy fiber zone; pcl, pyramidal cell layer; so, stratum oriens; sr, stratum radiatum; sub, subiculum. Scale bars **(A,B)** 500 μm, **(C–F)** 50 μm.

In European mole, weak NECAB2 staining is seen in the outer molecular layer, while the narrow middle molecular layer appears almost unstained. The deep dentate molecular layer is well-stained (Figure 3B). The outer and middle molecular layers stain with the same intensity in mouse. Staining increases slightly in the mouse deep molecular layer, but the contrast between the zones of the molecular layer is much less pronounced than in mole (Figure 3A). The subgranular zone is well-stained in mole (Figure 3B) but does not differ from the remainder of the hilus in mouse (Figure 3A). Like NECAB1, NECAB 2 is prominently expressed in large multipolar cells of the polymorphic cell layer (Figures 3A,B). They are present throughout the septotemporal axis in mole and mouse, but stained cells are smaller temporally in mouse.

In mouse and European mole, weak to moderate staining is observed in the deep pyramidal cells of CA3. The number of stained cells decreases proximally. In distal CA3, pyramidal cell staining is stronger than elsewhere (Figure 3C) and extends further into the apical pyramidal cell dendrites.

In the European mole, stained pyramidal cells are found predominantly in the deep CA1 pyramidal cell layer (Figure 3D), but not all deep CA1 pyramids are immunoreactive. NECAB2 is only found in a few, mostly proximal and deep CA1 pyramids in mouse (Figure 3A). Well stained cells are found in the superficial part of the cell layer of the proximal subiculum.

Notably, the staining of interneurons is, apart from a few dentate pyramidal basket cells, absent from all layers and in all hippocampal subfields in mole (Figure 3A). They are few in mouse, except for neurons along the border between the septal stratum radiatum and stratum lacunosum-moleculare.

We also observed NECAB2 staining of large hilar polymorphic cells in, e.g., domestic sheep (Figure 3E) and eastern rock sengi (Figure 3F).

#### 3.2.3 NECAB3

**Figure 4:**
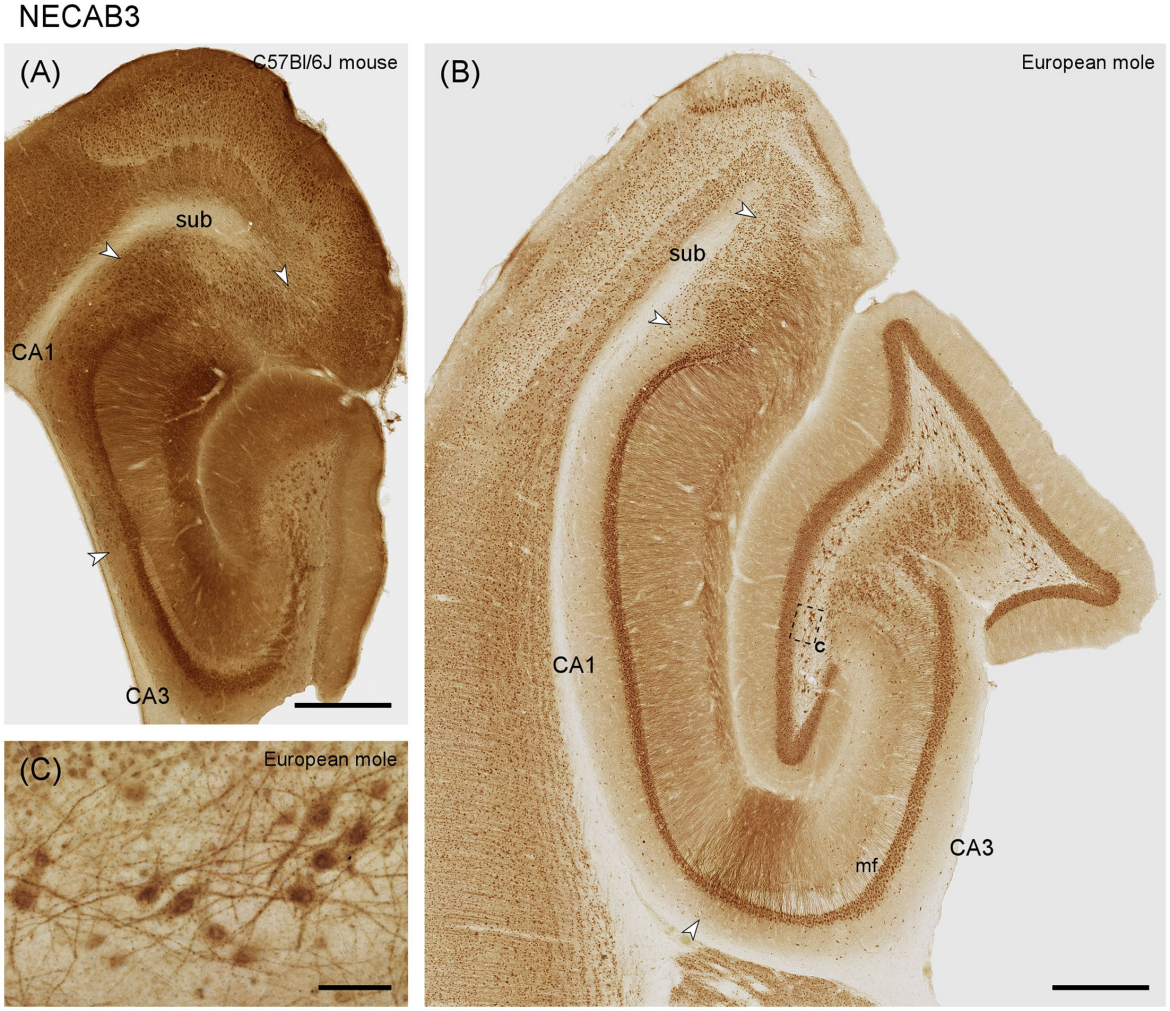
NECAB3 in the mid-septotemporal hippocampal region of the **(A)** C57BL/6 J mouse and **(B)** European mole. Arrowheads mark subfield boundaries. The dashed-bordered box marks the approximate locations of **(C)**, which was imaged in an adjacent section. **(C)** In the European mole, large multipolar neuron, almost certainly representing hilar mossy cells, stain strongly for NECAB3 in the hilar polymorphic cell layer with dark staining associated with the nuclei. mf, mossy fiber zone; sub, subiculum. Scale bars **(A,B)** 500 μm, **(C)** 50 μm.

NECAB3 distributes more similarly in mouse and mole hippocampus than NECAB1 and 2. The dentate molecular layer stains weakly in mole and moderately in mouse, with no differentiation along its radial axis (Figures 4A,B). Granule cells express NECAB3, which is also true for all other principal neuron populations of the hippocampus (Figures 4A,B). Once again, the multipolar cells in the hilar polymorphic cell layer and their proximal dendrites are well stained in the mole and mouse (Figure 4). In contrast to other NECABs, part of and sometimes the strongest staining is associated with the nucleus (Figure 4C) and, in large hilar multipolar neurons, in strongly staining granules. Positive interneurons are present in all layers and subfields of the mole hippocampus, and their staining is strongest in CA3 and stratum oriens of CA1. Interneuron staining is weaker in the mouse. Strongly staining apical dendrites, extending all the way to the hippocampal fissure, are present in distal CA3 and proximal CA1 of the mole (Figure 4B). Apical dendrites are also stained throughout CA1 and the subiculum of mole and mouse.

### 3.3 Distribution of other calcium-binding proteins in European mole

#### 3.3.1 Calbindin

**Figure 5:**
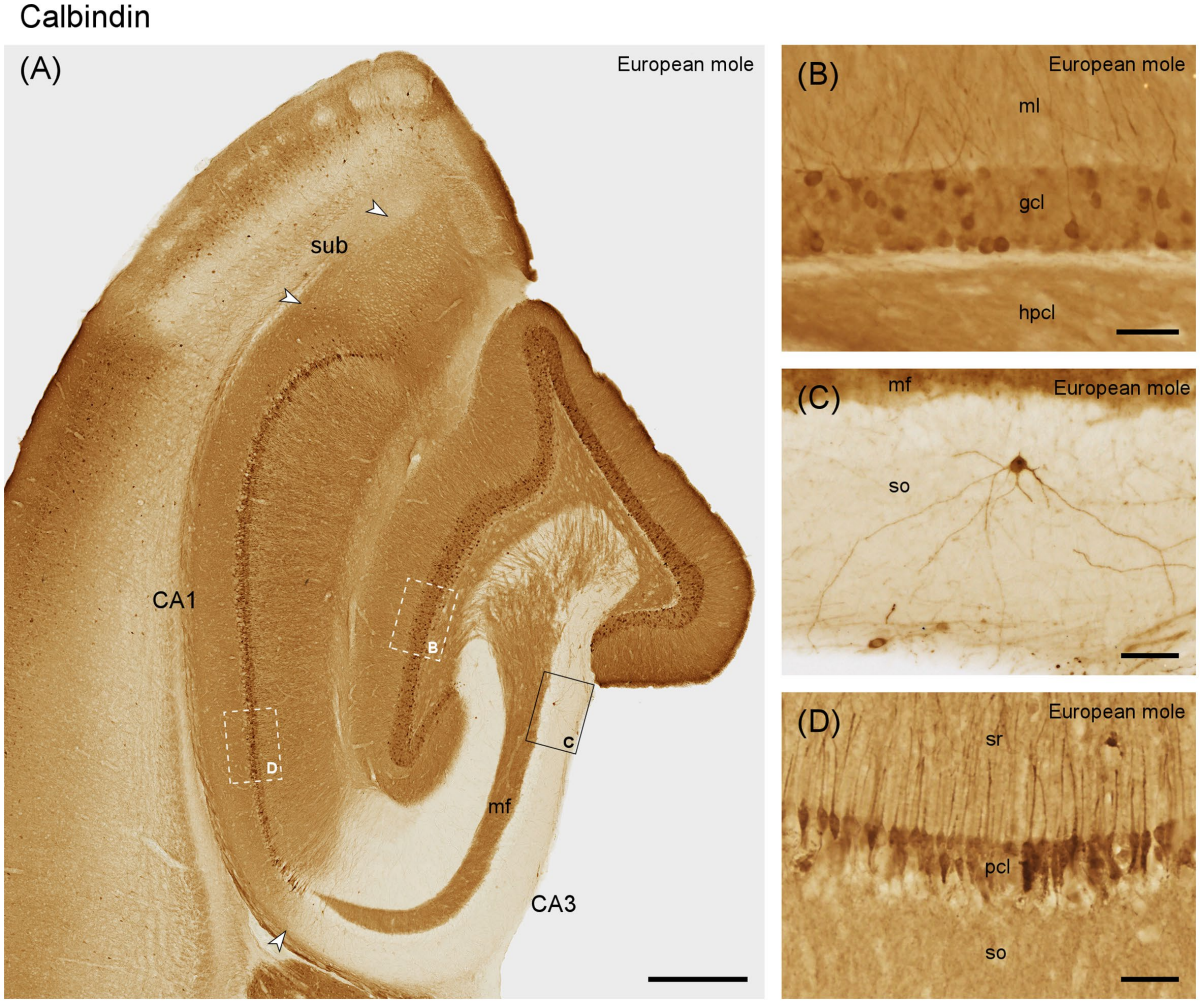
Calbindin in **(A)** the mid-septotemporal hippocampal region of the European mole. Arrowheads mark subfield boundaries. Box marks the location of **(C)**. Dashed-bordered boxes mark the approximate locations of **(B, D)**, which were imaged in adjacent sections. **(B)** All granule cells contain calbindin, but only a subpopulation is strongly immunoreactive. **(C)** Rare interneurons in the stratum oriens of CA3. **(D)** Calbindin-immunoreactive pyramidal cells in the superficial tier of the pyramidal cell layer. Unstained cell bodies of deep pyramidal cells are visible as voids in the deep pyramidal cell layer. gcl, granule cell layer; hpcl, hilar polymorphic cell layer; mf, mossy fiber zone; ml, molecular layer; pcl, pyramidal cell layer; so, stratum oriens; sr, stratum radiatum; sub, subiculum. Scale bars **(A)** 500 μm, **(B–D)** 50 μm.

The molecular layer is moderately stained (Figure 5A). Granule cells are generally immunoreactive, but only a subset shows strong immunoreactivity (Figures 5A,B). Immunoreactive fibers densely cover the entire hilar polymorphic cell layer. Except for the mossy fiber zone, CA3 layers appear almost unstained. CA3 pyramidal cells are calbindin negative, except for a few deep neurons close to the distal tip of the mossy fiber zone. These cells extend for a short distance into the deep, most proximal CA1 pyramidal cells layer (Figure 5A). Elsewhere in CA1, superficial, calbindin-positive pyramidal cells with dendrites extending into stratum oriens and radiatum are consistently present (Figures 5A,D). A few strongly stained very deep pyramids are present close to the subiculum. Few and weakly stained calbindin-positive neurons are limited to the superficial, proximal subicular cell layer.

Immunoreactive neurons that by their location or morphology may represent interneurons are rare throughout the hippocampus, although well-stained neurons were seen in adjacent cortical areas. When present in the hippocampus, they are typically seen in the hilar polymorphic cell layer and in adjacent parts of stratum radiatum and oriens of CA3 (Figure 5C).

#### 3.3.2 Calretinin

**Figure 6:**
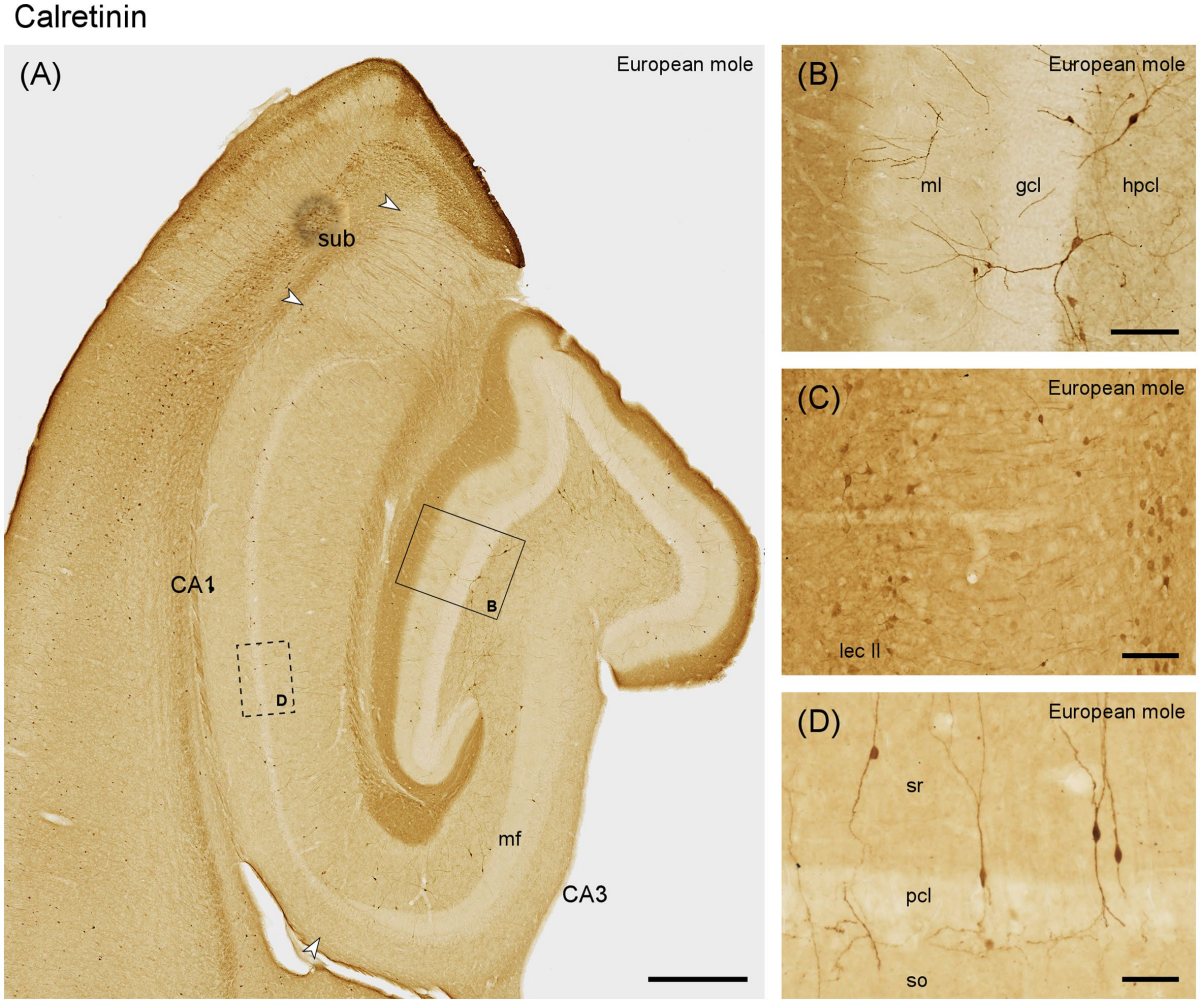
Calretinin in the **(A)** mid-septotemporal hippocampal region of the European mole. Arrowheads mark subfield boundaries. Box marks the location of **(B)**. The dashed-bordered box marks the approximate locations of **(D)**, which was imaged in an adjacent section. **(B)** Transect through the layers of the dentate gyrus. No staining suggestive of hilar mossy cells is visible. **(C)** Lateral entorhinal cortex of the mole, temporal to the section illustrated in A, with stained neurons in layers II (lec II). **(D)** Bipolar neurons, representing the most common type of calretinin-immunoreactive neurons in CA3, CA1 and subiculum, in stratum oriens of CA1. This type of cell is also commonly embedded in the CA1 pyramidal cell layer. They appear similar to NECAB1 immunoreactive bipolar neurons (Figure 2) but are more numerous. gcl, granule cell layer; hpcl, hilar polymorphic cell layer; mf, mossy fiber zone; ml, molecular layer; pcl, pyramidal cell layer; so, stratum oriens; sr, stratum radiatum; sub, subiculum. Scale bars **(A)** 500 μm, **(B,C)** 100 μm, **(D)** 50 μm.

The outer dentate molecular layer exhibits a rich, dark staining pattern (Figure 6A), which is likely to originate from immunoreactive cells in layer II of the entorhinal cortex (Figure 6C). Calretinin is absent from granule cells (Figures 6A,B). Small, round and strongly immunoreactive neurons are present in the middle and deep molecular layer (Figure 6B). Calretinin immunoreactive cells resembling mossy cells in number or distribution are absent along the entire septotemporal axis. Typically, 2 or 3 large multipolar calretinin-positive cells are found in the hilar polymorphic cell layer (Figures 6A,B) along the entire septotemporal axis. Their dendrites are seen branching within the hilar polymorphic cell layer, occasionally passing through the granule cell layer and branching also into the molecular layer. The staining of the outer dentate molecular layer extends into the stratum lacunosum-moleculare of CA3 and the deep part of stratum lacunosum-moleculare in CA1 (Figure 6A). Bipolar calretinin-positive neurons are found in stratum radiatum and the pyramidal cell layer of CA3 and CA1 (Figures 6A,D). They form the majority of the calretinin-immunoreactive interneurons in these fields, and their morphology and distribution correspond closely to that of NECAB1-immunoreactive bipolar cells. Their density increases in the distal stratum radiatum of CA3. In the subiculum, small, bipolar cells are present at the border between pyramidal cell layer and plexiform layer (Figure 6).

#### 3.3.3 Parvalbumin

**Figure 7:**
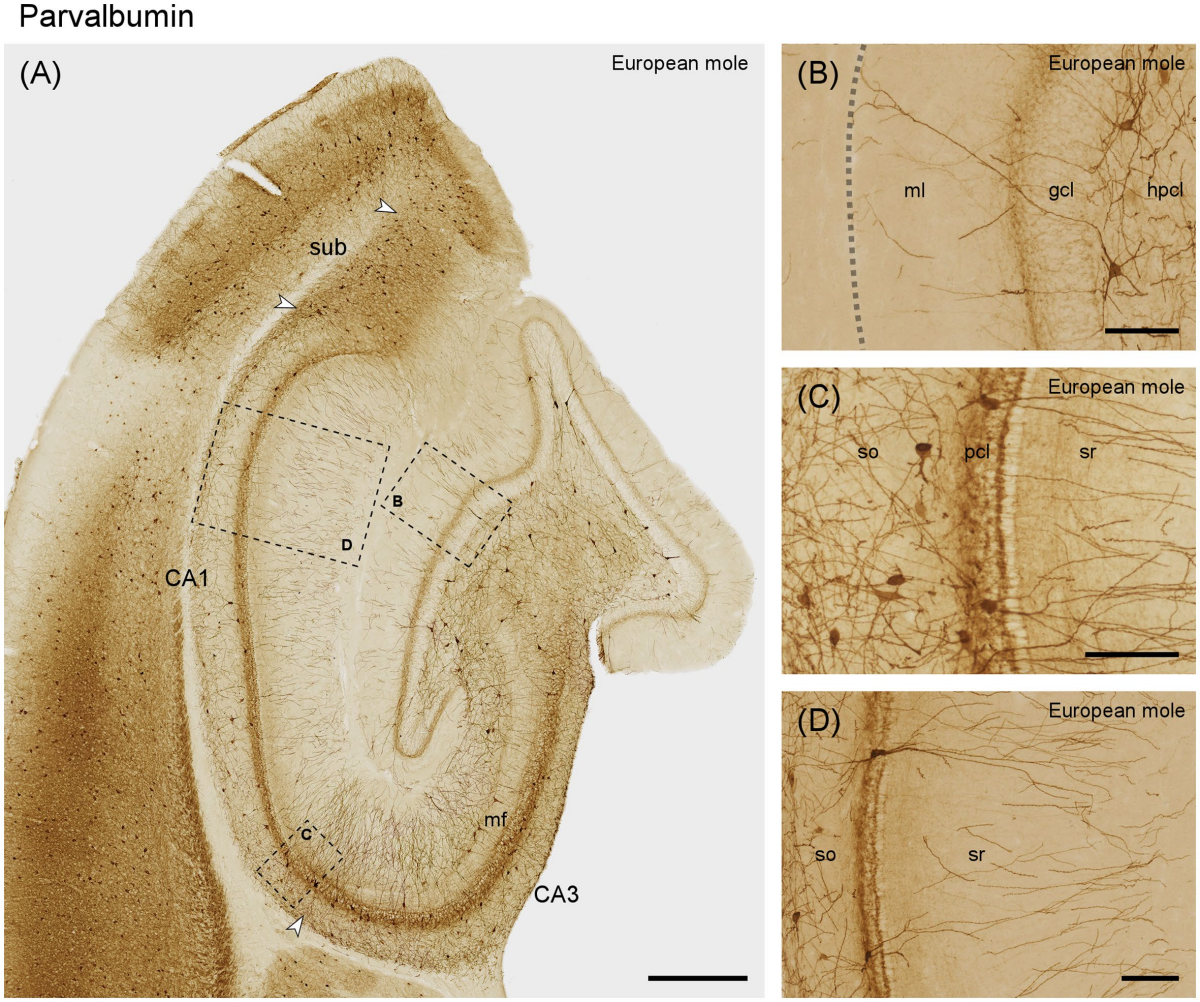
Parvalbumin in the **(A)** mid-septotemporal hippocampal region of the European mole. Arrowheads mark subfield boundaries. Dashed-bordered boxes mark the approximate locations of **(B–D)**, which were imaged in adjacent sections. **(B)** Transect though the layers of the dentate gyrus with interneurons in the hilar polymorphic cell layer. The dashed line marks the obliterated hippocampal fissure. **(C)** Proximal CA1 with frequent interneurons in the stratum oriens and embedded in the pyramidal cells layer. When the plane of the section is perpendicular to the plane of the pyramidal cell layer, superficial pyramids form a very regularly organized sublayer. At depth, it is delimited by a strongly parvalbumin-immunoreactive band, which may represent the Chandelier cell innervation of the axon initial segment of superficial pyramids. **(D)** Distal CA1 with immunoreactive neurons that morphologically may represent pyramidal basket and chandelier cells. Both proximally **(C)** and distally, immunoreactive neuronal somata are rare in layers superficial to the pyramidal cells. gcl, granule cell layer; hpcl, hilar polymorphic cell layer; mf, mossy fiber zone; pcl, pyramidal cell layer; so, stratum oriens; sr, stratum radiatum; sub, subiculum. Scale bars **(A)** 500 μm, **(B–D)** 100 μm.

Aside from dendrites originating from cells located in the hilus, most of the dentate molecular layer appears unstained (Figures 7A,B). A fine plexus of stained fibers is found immediately superficial to and between the most superficial granule cells (Figure 7B), which themselves are unstained. Large cells are observed at the border between the granule cell layer and subgranular layer. The dendrites of these cells branch locally but may also extend through the granule cell layer and across the full width of the molecular layer (Figure 7B). Additional large neurons with profusely branching dendrites are seen in the hilar polymorphic cell layer (Figure 7B). Most parvalbumin-positive cells of CA3 are found in the stratum oriens and within the CA3 pyramidal layer (Figure 7A). The dendrites of these cells often travel for long distances parallel to the pyramidal cell layer within stratum oriens. A few large immunoreactive cells are also found along the border between the mossy fiber zone and stratum radiatum (Figure 7A). Their dendrites branch profusely within stratum radiatum. Additional dendrites in stratum radiatum originate from neurons in the pyramidal cell layer. The pyramidal cells are unstained but embedded in a very dense plexus of fine fibers. The number of stained cells and dendrites appears to increase in the most distal CA3. This pattern continues for a short distance beyond the tip of the mossy fibers zone into proximal CA1 (Figures 7A,C). Thereafter, staining in superficial stratum oriens and stratum radiatum decreases sharply in the remainder of CA1 (Figures 7A,D). In the CA1 pyramidal cell layer, parvalbumin-positive cells are scarce, although a few are discernible in distal CA1 (Figures 7A,D). Similar to CA3, deep CA1 pyramids are embedded in a dense fiber plexus (Figure 7D). When the section plane passes perpendicular through the pyramidal cell layer, superficial pyramids form a very dense, almost unstained band. At depth, superficial pyramids are delimited by a narrow strongly immunoreactive band (Figure 7C), reminiscent of terminations of Chandelier cells. In the subiculum, parvalbumin immunoreactive cells are present along the deep cell layer. Superficially they are rare proximally but frequent distally. A dense plexus of fine fibers extends throughout the cell layer (Figure 7A).

## 4 Discussion

In this study, we present an overview of the distributions of NECAB1-3, parvalbumin, calretinin, and calbindin within the hippocampal subfields of the European mole. Our findings in the European mole confirm the regionally specific expression of the calcium-binding proteins calbindin, calretinin and parvalbumin as seen in other species, and provide novel observations on the distribution of the NECAB proteins. Of note is the consistent presence of NECABs within mossy cells of the hilar polymorphic cell layer.

NECAB1 and NECAB2 are primarily expressed in the brain, while NECAB3 is also expressed in muscle (Sugita et al., 2002). While the precise roles of these proteins are not fully understood, some studies have shed light on their potential functions. *In-vitro*, NECAB1 was found to be the only binding protein for the C_2_-domain of synaptotagmin, a synaptic vesicle protein involved in calcium-dependent exocytosis (Sugita and Südhof, 2000; Sugita et al., 2002). NECAB2 plays a role in the cell surface expression and function of the adenosine A_2A_ receptor (Canela et al., 2007), and interacts with mGlu5 glutamate receptor in hippocampal pyramidal cells (Canela et al., 2009). Moreover, NECAB2 was reported as downstream target of Pax6, a transcription factor indispensable for eye and brain development (Bernier et al., 2001). NECAB2 also influences striatal mitochondria function (Bueno et al., 2023). Furthermore, NECAB3, also known as XB51, was found to be involved in the regulatory system of the amyloid precursor proteins (Lee et al., 2000). NECAB1 and NECAB2 were also consistently colocalized with hippocampal CB1/CCK-positive interneurons in mice (Miczán et al., 2021).

In the European mole, we found NECAB distribution patterns that generally agree with observations in mice by us and others. In view of the time available since the lineages of mice and moles split, differences are minor. In that we also observed some of the traits shared by mouse and mole in other, taxonomcally diverse species, NECABs may be classed a rather conservative neuronal marker.

NECAB1 was staining a subset of granule cells in moles, while NECAB1 was absent from dentate granule cells in mice (Sugita et al., 2002; Zimmermann et al., 2013; Miczán et al., 2021). In addition, we found in the subiculum that proximal superficial cells in mice and deep cells in European mole were stained, which have not been described before. Sugita et al. (2002) found that NECAB1 expression in pyramidal cell to be specific to CA2. We did not observe NECAB1 expression in pyramidal cells on either side of the tip of the mossy fibers in mice or mole. Zimmermann et al. (2013) suggested that cross-reactivity with NECAB2 might have been responsible for the pattern observed by Sugita et al. (2002). Otherwise, staining patterns of NECAB1 in the European mole resembles that in mice, with the strongly stained hilar polymorphic cells as a prominent feature. The staining pattern of NECAB2 in European moles is by and large congruent with the observations in mice (Miczán et al., 2021). Hilar polymorphic cells are, again, NECAB2-positive in moles and mice, and NECAB2 was notably concentrated in the pyramidal cell layers on either side of the tip of the mossy fiber zone in moles and mice as shown here and by others (Zimmermann et al., 2013; Sanders et al., 2021), but not in rats (Canela et al., 2009).

For NECAB3 there is currently the least detailed histological information available. *In-situ* hybridizations in mouse indicated a scattered to ubiquitous NECAB3 signal in all neurons (Zimmermann et al., 2013; Girard et al., 2015), which we confirm here immunohistochemically in moles and mice. In contrast to NECAB1 and 2, the strongest staining of NECAB3 was associated with nuclei, similar to the localization of NECAB3 expression in cell cultures (Lee et al., 2000). Not only nuclei stained, but also the dendrites of all principal cells except granule cells in both species. Dendritic staining intensified in the CA2 region defined by both Lorente De Nó (1934) and Lein et al. (2005), particularly in the European mole. NECAB3 too was again strongly expressed in hilar polymorphic cells in both species.

In summary, all three NECABs analyzed here show distinct patterns of staining in the hippocampus. The specificity of NECABs for the CA2 subregion appears ambiguous, depending on the isoform of the NECABs, study or species. The consistent strong staining of hilar polymorphic cells along the entire septotemporal axis by all three NECAB’s is a promising feature of these proteins as this specificity is missing or restricted along the septotemporal axis for other calcium-binding proteins.

The expression of calbindin in hippocampal principal cells in European mole is in line with studies on mice (Fujise et al., 1995), rats (Jande et al., 1981; Baimbridge and Miller, 1982; Sloviter, 1989), mole-rats (Amrein et al., 2014), carnivores (Hof et al., 1996; Pillay et al., 2021), tree shrew (Keuker et al., 2003), monkeys (Seress et al., 1991) or humans (Sloviter et al., 1991; Lim et al., 1997). Deviating from this pattern, calbindin immunoreactivity is limited to the most distal and temporal CA1 in guinea pig, rabbit and sengi (Rami et al., 1987; De Jong et al., 1996; Slomianka et al., 2013). Instead, species with a less compact, stratified CA1 (e.g., carnivores and artiodactyla) can, in addition to the superficial tier, show strong calbindin immunoreactivity in deep, large pyramidal cells throughout the proximal-distal axis of CA1 (Hof et al., 1996; Pillay et al., 2021). Unusually, calbindin is widely expressed by deep CA3 pyramidal cells in naked mole-rats (Amrein et al., 2014). Interneuronal calbindin staining appears conservative across species as far as morphologies are concerned, but in European mole and sengi (Slomianka et al., 2013) these calbindin-positive interneurons are characterized by their overall scarcity and near absence from some layers.

Calretinin is rarely expressed in hippocampal principal cells. A notable exception are hilar mossy cells, which stain very weakly septally but gradually stronger toward the temporal hippocampus in laboratory mice (Liu et al., 1996; Blasco-Ibáñez and Freund, 1997) and other rodents (Murakawa and Kosaka, 1999; Maliković et al., 2023). From mid-septotemporal levels onwards, calretinin can serve as a marker for this cell type (Fujise et al., 1995). A similar pattern is found in other species (Seress et al., 2008; Maliković et al., 2023), although calretinin expression may be septotemporally “delayed” and only evident at the temporal pole of the dentate gyrus. Mossy cell-like staining and, consequently, calretinin staining of the commissural-associational, deep zone of the molecular layer remained absent, even at the temporal pole, in the European mole. It shares this trait with humans (Seress et al., 2008), African green monkeys (Seress et al., 1993) and laboratory rats (Freund et al., 1997; Jiang and Swann, 1997). Calretinin has been found in a subgroup of CA3 pyramidal cells in boar (Maliković et al., 2023), spiny rat (Fabene et al., 2001) and carnivores (Pillay et al., 2021) but was absent from this population in the European mole and other species (Maliković et al., 2023). The calretinin-positive projection from the entorhinal cortex to the superficial dentate molecular layer present in the mole has also been found in the guinea pig (Murakawa and Kosaka, 1999; Maliković et al., 2023). Interneuronal morphologies, consisting of mainly small ovoid or bipolar cells and fewer large multipolar cells are largely similar across species, although their regional density and distribution varies between species (Seress et al., 1993; Maliković et al., 2023).

Similar to our findings and previous observations in rats (Kosaka et al., 1987; Nitsch et al., 1990) and monkeys (Pitkänen and Amaral, 1993), hippocampal parvalbumin immunoreactivity is characterized by a remarkable robustness of the distribution and appearance of neurons with interneuronal morphologies and an equally robust absence of parvalbumin from hippocampal principal neurons. Similar observations have been made for the interneuronal expression in non-hippocampal cortical areas (Hof et al., 1999; Hof and Sherwood, 2005), although parvalbumin in the perforant pathway of the gerbil (Scotti and Nitsch, 1991; Bender et al., 2000) shows that also parvalbumin expression has some adaptive flexibility. The European mole hippocampus proper also contained a variety of parvalbumin immunoreactive cells and our observations generally confirm observations in mice (Jinno and Kosaka, 2006), rat (Kosaka et al., 1987), sengi (Slomianka et al., 2013), the African green monkey (Ribak et al., 1993) and human (Braak et al., 1991; Sloviter et al., 1991). It is noteworthy to observe that among various species, there is no discernible shift in the neuronal populations containing parvalbumin and calbindin (Kosaka et al., 1987; Nitsch et al., 1990; Ribak et al., 1990; Braak et al., 1991; Gulyás et al., 1991; Seress et al., 1991). It has been proposed that neuron populations engaged in feedback inhibitory circuits, like parvalbumin-and calbindin-positive cells, remain rather stable throughout phylogeny (Seress et al., 1993). In contrast, neuronal populations involved in feed-forward regulation of inhibition appear to undergo more dynamic changes (Seress et al., 1993).

One reason for us to investigate the European mole was the specialization of lifestyle that it shares with mole-rats. Mole-rats, in turn, share several histoarchitectural hippocampal traits not seen in other rodents, e.g., a small dentate gyrus, a very extensive CA3 and a distinct bilaminar CA1 (Amrein et al., 2014; van Dijk et al., 2016), which makes it tempting to speculate that these traits relate to their subterranean lifestyle. The hippocampus of the European mole provides a fine example that one should be wary of jumping to conclusions. It shows none of these traits. Another subterranean species that we have examined, the Golden mole (belonging to the order Afrosoricida), shows yet another pattern (unpublished observations and Bhagwandin et al., 2020). Evolutionary history and/or the necessities of the ecological or anatomical fine print that we cannot yet resolve have led to quite different hippocampal histoarchitectures despite grossly similar lifestyles. To these factors may belong diet (insectivorous mole versus herbivorous mole-rats) or somatosensory specializations in form of Eimer’s organs unique to the mole family, which consists of highly sensitive mechanoreceptors in the nose epithelium (Eimer, 1871; Catania, 1996).

We were also surprised by a histoarchitectural trait that is present in the European mole hippocampus, namely a differentiated reflected blade of CA3. The structural features and the possible function of this part of CA3 were briefly reviewed in Maliković et al. (2023). A well differentiated reflected blade of CA3 is present in the large brains of, e.g., some ungulate and primate species. It is also present in rabbits (Geneser, 1987), guinea pigs (Geneser-Jensen, 1972) and a few small rodents (Cavegn et al., 2013; Maliković et al., 2023), but with a simpler structure, more closely resembling the remainder of CA3. An exception is the reflected blade in the Eastern rock sengi (Slomianka et al., 2013), a small but highly polysensory, terrestrial species belonging to the Afrosoricida. Once again, there is no easy solution to relating hippocampal anatomy to gross features of the ecology of the species. While tools are at hand to investigate relations between phylogeny, ecology, and brain structure (Todorov et al., 2019; Schilder et al., 2020; Smaers et al., 2021; Kverková et al., 2022; Heuer et al., 2023), hippocampal traits are usually defined in too few species to apply them. The phylogenetic position of the European mole and its special ecology make it an interesting species to add to the pool that is becoming available for analysis.

## Data Availability

The original contributions presented in the study are included in the article/supplementary material, further inquiries can be directed to the corresponding author.
